# Orthogonally crosslinked gelatin methacryloyl microgels for in situ assembly of granular hydrogel scaffolds

**DOI:** 10.1002/btm2.70127

**Published:** 2026-04-28

**Authors:** Zaman Ataie, Nawaf Rajaa M. Alharbi, Angelo Roncalli Alves e Silva, Angie Castro, Arian Jaberi, Alexander Kedzierski, Aneesh Risbud, Roya Koshani, Amir Sheikhi

**Affiliations:** ^1^ Department of Chemical Engineering The Pennsylvania State University Pennsylvania USA; ^2^ Department of Biomedical Engineering The Pennsylvania State University Pennsylvania USA; ^3^ Núcleo de Biologia Experimental Universidade de Fortaleza Fortaleza Ceará Brazil; ^4^ Department of Chemistry The Pennsylvania State University Pennsylvania USA; ^5^ Department of Neurosurgery, College of Medicine The Pennsylvania State University Hershey Pennsylvania USA; ^6^ Huck Institutes of the Life Sciences The Pennsylvania State University Pennsylvania USA

**Keywords:** biomaterial, granular hydrogel, in situ fabrication, porous hydrogel, regenerative medicine

## Abstract

Gelatin methacryloyl (GelMA) microgels formed via physical crosslinking dissolve at the physiological temperature, hindering their translational use for the in situ fabrication of granular hydrogel scaffolds (GHS). To overcome this challenge, we present a dual crosslinking strategy that combines Schiff base chemistry for GelMA microgel stabilization with subsequent free‐radical photocrosslinking to form inter‐microgel covalent bonds. This technology yields stable microgels (SμG) that may be packed and photochemically interlinked in situ to form robust granular hydrogel scaffolds (GHS^SμG^) under physiological conditions. The resulting GHS^SμG^ support high cell viability and proliferation and have pore architecture and interconnectivity, as well as mechanical properties comparable to scaffolds fabricated using physically crosslinked GelMA microgels. This work is a step forward in developing translational granular biomaterials for the in situ tissue regeneration.

## Graphical abstract

We present a dual crosslinking strategy that stabilizes gelatin methacryloyl (GelMA) microgels at the physiological temperature while enabling their in situ assembly and covalent interlinking. This technology overcomes a key translational barrier to GelMA granular hydrogel scaffold formation on living tissues, yielding injectable, light‐curable protein‐based porous biomaterials for regenerative applications.

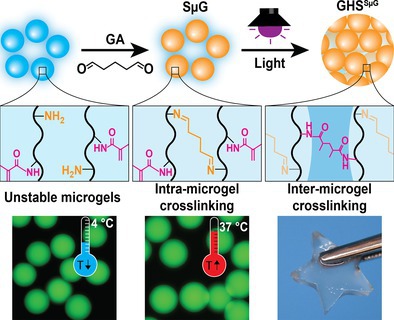

**Translational Impact Statements**

Porous granular hydrogel scaffolds (GHS) comprising interlinked microgels enhance tissue repair by promoting cell infiltration, vascularization, and metabolite transport through their interconnected macropores; however, physically crosslinked gelatin‐based microgels are thermally unstable and dissolve at body temperature, limiting clinical in situ use. We chemically address this challenge by developing stable gelatin methacryloyl microgels that form robust GHS at the physiological temperature. This work introduces a translational biomaterial platform that eliminates the need for cold‐chain handling, which may provide a solution for the in situ regeneration of irregular soft tissue defects.

## 1 INTRODUCTION

Despite widespread use in biomedical applications, bulk hydrogels formed via polymer crosslinking have nanoporous structures, which restrict metabolite diffusion and immediate cell infiltration. Granular hydrogel scaffolds (GHS),^1^ composed of packed hydrogel microparticles (microgels),^2^, ^3^ have emerged to address this limitation by providing interconnected cell‐scale pores, facilitating rapid cell infiltration, proliferation, and efficient nutrient transport.^4^, ^5^ Gelatin methacryloyl (GelMA) GHS are fabricated via packing GelMA microgels in an aqueous solution, followed by photo‐initiated free‐radical crosslinking.^6^ To synthesize GelMA, a subset of gelatin's free amine groups are reacted with methacrylic anhydride (MAA), mainly introducing methacryloyl (MA) functional groups while preserving some of native collagen‐derived sequences, such as arginylglycylaspartic acid (RGD) motifs and matrix metalloproteinase (MMP)‐responsive sites.^7^, ^8^ Via these bioactive features, GelMA GHS support cell adhesion and undergo enzymatic degradation, while their porous structure facilitates cell ingrowth, migration, and proliferation.^6^ In addition to GelMA biopolymer, other natural and synthetic polymers, such as chitosan,^9^ alginate,^10^ hyaluronic acid,^11^ and polyethylene glycol (PEG),^12^ have been used to fabricate microgels for GHS fabrications, providing complementary chemistries and crosslinking modalities to gelatin‐based systems.^2^ The packing conditions of microgels in GelMA GHS influence scaffold characteristics, such as pore size, geometry, and mechanical properties, significantly impacting cell activities.^13^ Recent advances in GelMA GHS applications include three‐dimensional (3D) bioprinting,^14^ cancer research,^15^ wound healing,^16^ and patterned neovascularization through integration with microsurgical techniques.^17^, ^18^

Conventionally, GelMA GHS are fabricated through sequential physical and chemical crosslinking.^19^ Initially, GelMA microgels are physically crosslinked via temperature‐driven gel formation below the sol–gel transition temperature, temporarily stabilizing their structure. Physically stabilized GelMA microgels are subsequently packed via centrifugation and chemically crosslinked through free‐radical photopolymerization, forming inter‐ and intra‐microgel covalent bonds.^17^ However, physically crosslinked GelMA microgels dissolve at the physiological temperature (37°C) because of their thermally reversible network, limiting in situ translational applications in physiological environments.^6^ Recent studies have addressed this issue by partially photocrosslinking GelMA microgels prior to scaffold formation, providing stability and allowing residual MA groups to participate in subsequent inter‐microgel bonding for GHS formation.^20^, ^21^ We have recently conducted a comprehensive optimization of this method, focusing on balancing microgel stability and residual MA groups for inter‐microgel covalent bond formation, establishing conditions for predictable microgel stiffness and effective assembly.^21^ Despite these advancements, decoupling microgel stabilization and GHS formation methods remains an unmet need for developing injectable GelMA microgels that can form mechanically robust porous GHS on tissues.

Gelatin and GelMA gels are commonly formed through four crosslinking approaches: (i) physical methods^22^, ^23^; (ii) chemical methods, such as using EDC/NHS (1‐ethyl‐3‐(3‐dimethylaminopropyl)carbodiimide/*N*‐hydroxysuccinimide),^24^ formaldehyde,^24^ glutaraldehyde (GA),^24^, ^25^, ^26^ genipin,^25^ and free‐radical (photo)crosslinking^8^, ^16^; (iii) enzymatic methods, such as microbial transglutaminase^25^, ^27^; and (iv) crosslinking methods that combine the above techniques.^6^, ^17^, ^28^ In this study, we introduce a dual crosslinking approach using a Schiff base reaction to chemically stabilize GelMA microgels without compromising MA groups, followed by the photocrosslinking of MA groups to form GHS. This strategy involves the initial stabilization of GelMA microgels using GA, enabling stable Schiff base intra‐microgel crosslinks that prevent microgel dissolution at physiological conditions. Subsequently, the chemically stabilized microgels undergo photocrosslinking, establishing robust inter‐microgel covalent bonds. Our dual crosslinking technique enables the in situ formation of GelMA GHS under physiological conditions without compromising scaffold mechanical integrity, porosity, and biological functionality. This approach may expand the in situ applications of GelMA GHS for regenerative engineering, where rapid and stable porous scaffold formation on tissues is required.

## 2 EXPERIMENTAL

### 2.1 Materials

Gelatin powder (Type A, ~300 g Bloom, from porcine skin), methacrylic anhydride (MAA, contains 2000 ppm topanol A as inhibitor, ≥98%), lithium phenyl‐2,4,6‐trimethylbenzoylphosphinate (LAP), fluorescein isothiocyanate‐dextran (FITC‐dextran, average molecular weight ~2 MDa), 1*H*,1*H*,2*H*,2*H*‐perfluoro‐1‐octanol (PFO), deuterium oxide (D_2_O, 99.9 atom % D), and GA (25% aqueous solution) were purchased from MilliporeSigma (MA, USA). Milli‐Q water purification system, used to produce ultrapure water (electrical resistivity ~18.2 MΩ cm at 25°C), was provided by Millipore Corporation (MA, USA). Vacuum filtration unit (pore size = 0.2 μm) was purchased from VWR (PA, USA). Novec™ 7500 Engineered Fluid was purchased from 3M (MN, USA). Pico‐Surf (2% w/w in Novec™ 7500) was purchased from Sphere Fluidics (Cambridge, UK). Tygon® tubing (inner diameter = 0.51 mm, wall thickness = 0.80 mm) was purchased from Saint‐Gobain (NJ, USA). Syringes (10 mL, Luer‐Lok™) were purchased from BD Biosciences (NJ, USA). Fetal bovine serum (FBS) and antibiotic/antimycotic solution (10,000 U mL^−1^ penicillin G, 10,000 μg mL^−1^ streptomycin, 25 μg mL^−1^ amphotericin B) were purchased from Cytiva (MA, USA). Dulbecco's phosphate buffered saline (DPBS, liquid), Dulbecco's Modified Eagle's Medium (DMEM, high glucose, with L‐glutamine and sodium pyruvate), collagenase Type I (295 Units mg^−1^), and trypsin‐ethylenediaminetetraacetic acid (EDTA) solution (0.25%) were purchased from Gibco (MA, USA). Paraformaldehyde (PFA) was purchased from Thermo Scientific (IL, USA). T‐75 cell culture flasks, 24‐well non‐treated culture plates, 48‐well non‐treated culture plates, and centrifuge tubes (15 and 50 mL) were purchased from Celltreat Scientific Products (MA, USA). Black polystyrene 96‐well flat clear bottom microplates were purchased from Corning (NY, USA). Live/Dead cell imaging kit (488/570) containing Calcein acetoxymethyl ester (Calcein AM) and BOBO‐3 iodide, 4′,6‐diamidino‐2‐phenylindole (DAPI), Alexa Fluor™ 488 phalloidin, and PrestoBlue HS (high sensitivity) cell viability reagent were purchased from Invitrogen (MA, USA).

### 2.2 GelMA synthesis

GelMA was synthesized following an established protocol.^19^ Briefly, a 10% w/v gelatin solution in 100 mL of DPBS was prepared at 50°C under continuous stirring at 200 rpm. For a high, medium, or low degree of MA substitution (*D*_S_), 8.00, 1.25, or 0.25 mL of MAA was added dropwise, while maintaining the biphasic solution at a constant temperature of 40°C. The reaction was carried out for 2 h while being shielded from light. The solution was diluted with DPBS (1:1 v/v) to end the reaction, which was then dialyzed against ultrapure (Milli‐Q) water at 40°C for 10 days and filtered using a 0.2 μm vacuum filtration system, followed by freezing at −80°C for at least 2 days prior to lyophilization (collector temperature approximately −80°C and vacuum pressure ~0.04 mbar) using a Labconco FreeZone 4.5 L Benchtop freeze dryer (Labconco, MO, USA) to yield solid GelMA biopolymers with varying *D*_S_.

### 2.3 Droplet formation

GelMA droplets were fabricated using a high‐throughput step‐emulsification microfluidic device, as described previously.^17^ The dispersed phase consisted of a 10% w/v GelMA solution prepared in DPBS, and the continuous oil phase was the Novec™ Engineered Fluid, containing 2% Pico‐Surf surfactant. The setup was maintained at around 40°C using a space heater to ensure GelMA solubility throughout the process. To form droplets, the dispersed and continuous phases were loaded into 10 mL syringes, then introduced to the microfluidic chip using syringe pumps (PHD 2000, Harvard Apparatus, MA, USA) via Tygon® tubing (inner diameter: 0.51 mm, wall thickness: 0.80 mm) at controlled flow rates of 40 and 80 μL min^−1^ for the dispersed (GelMA) and oil phases, respectively. Droplets were generated via dispersed phase expansion into the continuous oil phase. The resulting droplets were collected in 15 mL centrifuge tubes and stored at 4°C overnight to yield physically crosslinked microgels.

### 2.4 Microgel stabilization via a Schiff base reaction

The oil and surfactant surrounding the physically crosslinked microgels were removed by adding a 1:1 volume ratio of 20% v/v PFO in the Novec™ oil and precooled to 4°C. The mixture was subsequently spun at 300 × *g* for 15 s to pellet the microgels, then the oil was removed. The microgels were resuspended in an equal volume of 4°C DPBS. The suspension was vortexed, then centrifuged again at 300 × *g* for 15 s. The washing step with DPBS was repeated twice to ensure complete oil removal. The washed microgels were resuspended in DPBS, vortexed, and packed via centrifugation at 3000 × *g* for 15 s. The supernatant was discarded, and the microgels were transferred into a 0.15% w/v GA solution in DPBS, precooled to 4°C. The suspension was continuously stirred at 200 rpm using a magnetic stirrer to maintain homogeneity and prevent microgel aggregation. The reaction was carried out at 4°C for 30 min, allowing intra‐microgel Schiff base formation to stabilize the microgels while preventing inter‐microgel attachment. To terminate the reaction, DPBS (four times the reaction volume, 4°C) was added to dilute the GA solution. The microgels were centrifuged at 300 × *g* for 5 min, and the supernatant was discarded. To ensure the complete removal of unreacted GA, fresh DPBS was added at a 1:1 volume ratio, and the washing step was repeated at least five times. The product of this step was stable microgels (SμG), which were stored at 4°C in DPBS until further use.

### 2.5 GHS and GHS^SμG^ fabrication

SμG suspensions were transferred into 1.7 mL microcentrifuge tubes in 1 mL aliquots and packed by centrifugation at 300 × *g* for 15 s. The supernatant was discarded, and a 0.1% w/v LAP photoinitiator (PI) in DPBS was added at a 1:2 volume ratio relative to the packed microgels. The microgel suspension was thoroughly mixed using a vortex mixer and centrifuged again at 300 × *g* for 15 s. After discarding the supernatant, a fresh PI solution (0.1% w/v) was added, and the suspension was vortexed to ensure uniform mixing. The microgel suspension was then incubated at room temperature for 1 h. GHS made from SμG (GHS^SμG^) were assembled by packing the stable building blocks via centrifugation at 3000 × *g* for 15 s. After discarding the supernatant, the packed microgels were transferred into laser cut acrylic molds using a positive displacement pipette (MICROMAN E M100E, Gilson, WI, USA) and exposed to light (395–400 nm, 15 mW cm^−2^) for 2 min to form covalently crosslinked GHS^SμG^. Conventional GHS were formed as described in our previous publication.^19^ Briefly, physically crosslinked microgels were washed with 20% v/v PFO in the Novec™ oil (1:1 volume ratio) at 4°C and centrifuged at 300 × *g* for 15 s so that the oil and surfactant could be removed. Then, the microgels were washed twice with the PI solution and centrifuged at 300 × *g* for 15 s, followed by packing via centrifugation at 3000 × *g* for 15 s. They were then transferred to the mold and exposed to the light, similar to the GHS^SμG^ fabrication process, yielding GHS. All washing and crosslinking steps of conventional GHS were conducted at 4°C (cold‐chain conditions) to preserve the stability of physically crosslinked GelMA microgels.

### 2.6 Proton nuclear magnetic resonance spectroscopy

GelMA *D*_S_ was quantified using proton nuclear magnetic resonance (^1^H NMR) spectroscopy. Approximately, 40 mg of lyophilized GelMA was dissolved in 1 mL of D_2_O and incubated at 37°C for 2 h to ensure complete dissolution. Samples were analyzed using a 400 MHz Bruker NEO NMR spectrometer (Bruker, MA, USA) at the Pennsylvania State University NMR facility. Peaks corresponding to the vinyl protons (~5.3–6.0 ppm) of MA groups, lysine methylene protons (~2.8–3.2 ppm), and reference peaks from phenylalanine (~7.0–7.6 ppm, used for normalization) were integrated using TopSpin (version 4.2.0, Bruker, MA, USA). The $D_{S}$ was calculated using the following equation^29^:

(1) $$D_{S}=\left(1-\frac{\text{Normalized area under the lysine methylene proton peaks in GelMA}}{\text{Normalized area under the lysine methylene proton peaks in gelatin}}\right)$$

### 2.7 Rheological characterizations

Oscillatory strain sweep tests were performed on cylindrical samples (diameter = 8 mm and height = 1 mm) to determine dynamic moduli, namely oscillatory shear storage modulus (*G*′) and loss modulus (*G″*). The samples were incubated in DPBS for at least 1 h at room temperature prior to each test. Rheological measurements were performed using a rheometer (TA Instruments, DE, USA), equipped with sand‐blasted parallel plates (25 mm bottom, 8 mm top) set at a 1 mm gap. Sample temperature was maintained at 25°C or 37°C throughout the tests. Oscillatory strain sweeps (0.1%–1000% strain) were conducted to determine *G*′ and *G″* at constant frequency (1 rad s^−1^).

### 2.8 Compression test

GHS compressive modulus was measured using a mechanical tester (Instron 5943, MA, USA). Cylindrical samples (8 mm diameter × 1 mm height) were compressed at a rate of 1 mm min^−1^, and the corresponding stress–strain data were used to calculate compressive modulus by measuring the slope in the linear elastic region at ~5% to 15% of strain.

### 2.9 Pore analysis

The 3D pore architecture of GelMA GHS^SμG^ scaffolds was analyzed using the segmentation and image processing of *Z*‐stacked confocal microscopy images. Confocal images were acquired using a Leica STELLARIS 5 White Light Laser scanning confocal microscope (Leica Microsystems, Germany). To visualize the void spaces among microgels, a high molecular weight FITC‐dextran (Mw ~2 MDa, 15 μM in DPBS) was used as a fluorescent agent. This dye was selected due to its ability to diffuse through the interconnected GHS^SμG^ pore network while being excluded from individual microgels. Fluorescence excitation was performed at 490 nm, and emission was collected at 500–580 nm. Confocal microscopy images were acquired using a 10x objective lens (numerical aperture = 0.45) with a *Z*‐depth of 74 μm to capture volumetric pore structures. The acquired images were processed in Avizo 3D software (version 2023.1.1, Thermo Fisher Scientific, MA, USA). The image stacks were first thresholded and binarized to segment the porous architecture. The void fraction was measured from the binarized image using the software's built‐in function. Automated computational segmentation was then performed using a Chamfer‐based conservative separation algorithm to distinguish connected microgel structures while preserving pore boundaries. Following segmentation, a pore network model (PNM) was constructed to assess scaffold interconnectivity, with color‐coded radii representing size distributions. Quantitative pore characteristics, including pore volume distribution, equivalent pore diameter, coordination number, and channel length between pores, were extracted from the PNM.

### 2.10 GHS enzymatic degradation

GHS and GHS^SμG^ were fabricated as previously described (diameter = 4 mm, height = 1 mm), followed by incubation in 1 mL of a collagenase solution in DPBS at a final concentration of 5 U mL^−1^ in individual wells of a 48 well‐plate. The samples were maintained at 37°C and imaged in real time over 42 h using a Leica DMi8 THUNDER microscope (Leica Microsystems, Germany). The area of GHS and GHS^SμG^ was measured at varying times using ImageJ (Fiji, version 1.54f, NIH, MD, USA), as shown in Figure S1, and normalized based on the following equation:

(2) $$\text{Normalized area}=\frac{\text{Area of}\;\mathrm{GHS}\;\mathrm{at}\;a\;\text{given timepoint}}{\;\text{Initial area of}\;\mathrm{GHS}\;\mathrm{at}\;\text{time}=0\;h}$$

### 2.11 Cell culture

NIH/3T3 murine fibroblast cells were cultured in DMEM supplemented with 10% v/v FBS and 1% v/v antibiotic. The cells were maintained in a standard cell culture incubator (Eppendorf C170i, Hamburg, Germany) at 37°C under a humidified atmosphere containing 5% v/v carbon dioxide (CO_2_). The culture media were refreshed every other day, and the cells were passaged at ~80% confluency. To passage cells, they were rinsed with sterile DPBS, followed by treatment with a 0.25% w/v trypsin–EDTA solution for 5 min at 37°C to detach the cells. Trypsin activity was neutralized by adding an equal volume of culture media, then the cell suspension was pelleted (300 × *g*, 5 min). The supernatant was removed, and the cell pellet was gently resuspended in fresh complete media for reseeding or experimental use.

### 2.12 In vitro culture in GHS and GHS^SμG^

GelMA GHS and GHS^SμG^ were placed in non‐treated 24‐well multiwell plates filled with DPBS, including penicillin–streptomycin (200 U mL^−1^). The DPBS was then aspirated, and 5 μL of NIH/3T3 cells (10^7^ cells mL^−1^) were pipetted on top of the GHS (height = 1 mm and diameter = 4 mm) and incubated for 30 min to ensure cell adhesion to the microgels, followed by the addition of supplemented DMEM and culturing the cells in the cell culture incubator. The media were refreshed daily.

### 2.13 Cell metabolic activity

The PrestoBlue HS cell viability kit was used to assess the metabolic activity of cells cultured within GHS and GHS^SμG^ over time. A 1:10 volume ratio of PrestoBlue stock solution was diluted in serum‐free DMEM to prepare the working solution. Each scaffold was treated with 1 mL of the PrestoBlue working solution, followed by incubation at 37°C under a 5% CO_2_ atmosphere for 4 h. Then, 100 μL of each supernatant was transferred to a 96‐well microplate for fluorescence quantification using a Tecan Infinite M Plex microplate reader (Männedorf, Switzerland) at 530 nm excitation and 590 nm emission wavelengths.

### 2.14 Cell viability

Cell viability was evaluated using a dual fluorescent staining protocol with Calcein AM for live cell staining and BOBO‐3 iodide for dead cell visualization. Briefly, 1 mL of a Calcein AM solution was added to 1 μL of BOBO‐3 iodide and mixed to prepare a stock solution. The stock solution was diluted by adding 1 mL of DPBS to prepare the staining solution. To perform the assay, 1 mL of staining solution was added to each scaffold. Then, samples were incubated at ambient temperature for 30 min while protected from light. Following incubation, scaffolds were gently rinsed with DPBS to remove unbound dye and imaged using a Leica DMi8 THUNDER Imager 3D Cell Culture microscope (Leica Microsystems, Germany). The live cell channel was set to excitation/emission wavelengths of 470/510 nm, and the dead cell channel was set to excitation/emission wavelengths of 550/610 nm. The total area of cells was determined by adding the live and dead cell counts from the respective fluorescence channels. Quantitative analysis was conducted using ImageJ (Fiji, version 1.54f, NIH, MD, USA)^30^ based on the following equation:

(3) $$\text{Cell viability}\;\left(\%\right)=\frac{\text{Area of Calcein}\;\mathrm{AM}}{\text{Area of Calcein}\;\mathrm{AM}+\text{Area of BOBO‐}3\;\mathrm{iodide}}\times 100$$

### 2.15 Cell morphology

For cell morphology characterization, actin/DAPI staining was performed. On days 1, 7, and 14 post‐seeding, scaffolds were fixed using PFA (4% v/v) for 30 min, followed by three DPBS washes. Samples were then permeabilized with 0.3% Triton X‐100 for 15 min and incubated with Alexa Fluor™ 488 phalloidin (1:400 in DPBS) for 1 h at room temperature. After two DPBS washes, samples were incubated with DAPI (1:1000 in DPBS) for 5 min at room temperature. Actin and nuclei were visualized using Alexa Fluor™ 488 phalloidin and DAPI, respectively. Stained samples were imaged using a Leica DMi8 laser scanning confocal microscope, equipped with the STELLARIS 5 White Light Laser (Leica Microsystems, Germany). Actin area was measured using ImageJ (FIJI, version 1.53t).^30^

### 2.16 Statistical analysis

Data was acquired with at least four repeats. Results are expressed as mean ± standard deviation. Data was plotted and analyzed using GraphPad Prism (version 10.4.1). Student's *t*‐test, as well as ordinary one‐way or two‐way analysis of variance (ANOVA) were performed, followed by Tukey's post‐hoc test for multiple group comparison. *p*‐values < 0.05 were considered significant. The level of significance was shown with *p < 0.05, **p < 0.01, ***p < 0.001, and ****p < 0.0001.

### 2.17 Ethics approval statement

No animals or ex vivo tissues were used.

## 3 RESULTS AND DISCUSSION

### 3.1 Stabilization of GelMA microgels via a Schiff base reaction, followed by scaffold formation

**FIGURE 1:**
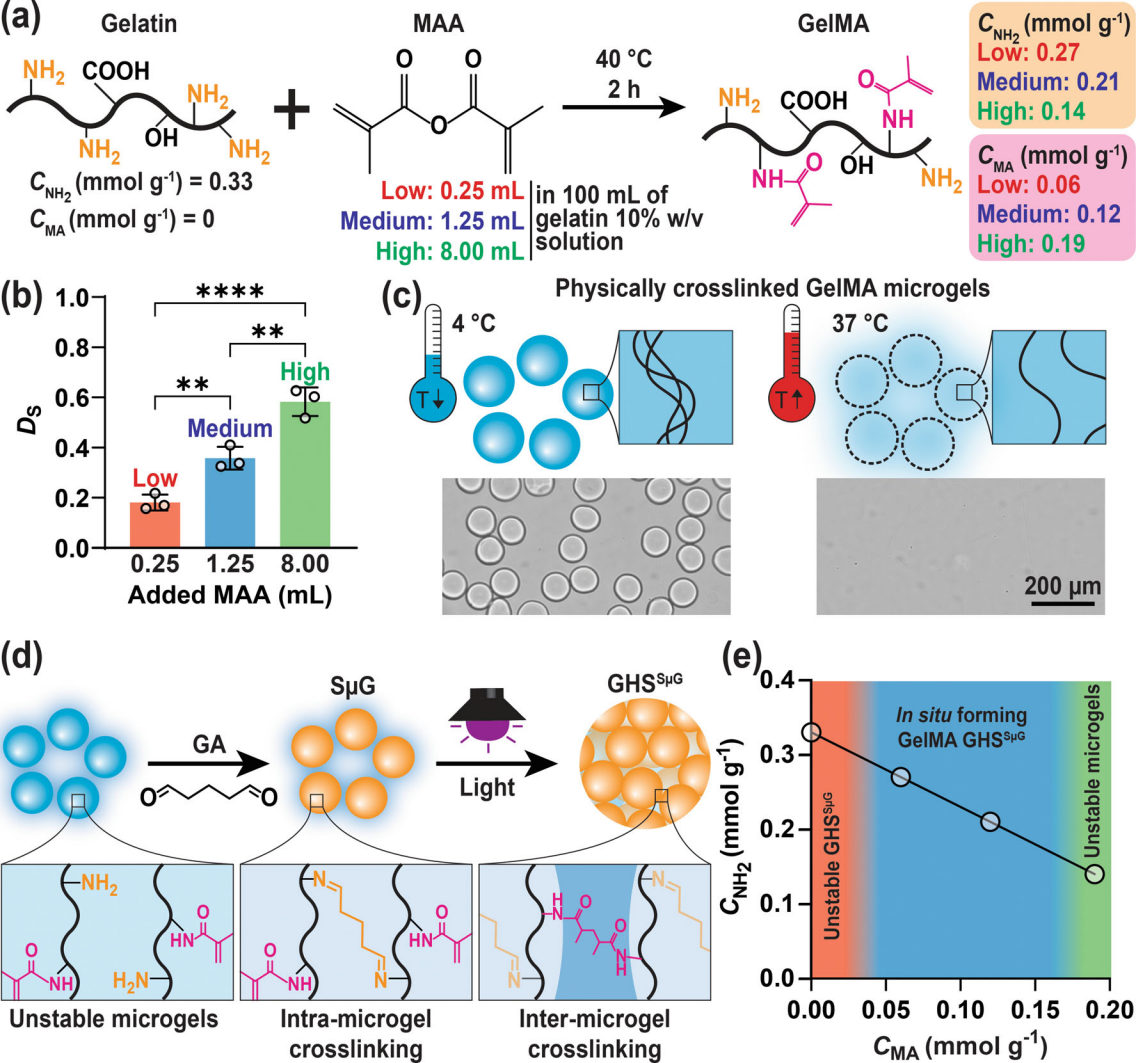
Stabilization of GelMA microgels via a Schiff base reaction, followed by scaffold formation. (a) Chemical synthesis of GelMA by reacting gelatin with MAA at varying concentrations. During this reaction, a hydrogen atom from a primary amine group in gelatin is substituted with a methacrylamide group. (b) Degree of substitution ($D_{s}$) for low, medium, and high GelMA. (c) Physically crosslinked GelMA microgels are stable at temperatures below the sol–gel transition temperature and dissolve at the physiological temperature (37°C), which renders them unsuitable for in situ GHS fabrication. (d) GHS formation involves two steps. First, physically crosslinked microgels, unstable at the physiological temperature, undergo a Schiff base reaction with glutaraldehyde (GA) to yield stable microgels (SμG), which do not dissolve at the physiological temperature. Then, SμG are packed and photocrosslinked to form inter‐microgel covalent bonds, resulting in robust GHS^SμG^. (e) Diagram showing the relationship between free amine ($C_{NH_{2}}$) and methacryloyl ($C_{\mathrm{MA}}$) contents, highlighting the region suitable for in situ GHS^SμG^ formation. One‐way analysis of variance is performed, followed by Tukey's post‐hoc multiple comparison test (**p < 0.01, and ****p < 0.0001).

GelMA biopolymer is synthesized by adding 0.25, 1.25, or 8.00 mL of MAA to 100 mL of a gelatin solution (10% w/v), yielding low, medium, or high *D*_S_, respectively (Figure 1a). This methacrylation reaction introduces two classes of photocrosslinkable vinyl groups: (i) methacrylamides formed when ε‐primary amines on lysine and hydroxylysine residues are acylated, and (ii) methacrylates formed by hydroxyl‐containing residues such as serine, threonine, and hydroxyproline.^7^, ^31^ Quantitative structural analyses of GelMA prepared in DPBS show that methacrylamides constitute >90% of all incorporated MA groups, confirming that photocrosslinkable sites are predominantly amide‐derived.^31^ *D*_S_ indicates the substitution of a hydrogen atom in primary amines by an MA group, calculated based on the ^1^H NMR results, as shown in Figure S2. The average primary amine content ($C_{NH_{2}}$) in non‐reacted gelatin (*D*_S_ = 0%) is ~0.33 mmol g^−1^,^29^, ^32^, ^33^ which decreases to 0.27, 0.21, or 0.14 mmol g^−1^ for low, medium, or high $D_{S}$ (18 ± 3%, 36 ± 5%, or 58 ± 6%), respectively (Figure 1b). Concurrently, average MA content ($C_{\mathrm{MA}}$) increases post‐methacrylation to 0.06, 0.12, or 0.19 mmol g^−1^ for low, medium, or high *D*_S_, respectively.

GelMA microgels are physically crosslinked via the partial recovery of protein structures,^34^, ^35^ remaining stable at 4°C but dissolving at physiological temperature (37°C), as presented in Figure 1c. Such physical crosslinking stabilizes microgels, enabling GHS fabrication at temperatures below the sol–gel transition temperature.^6^ GA is used to render these microgels stable at physiological temperature for on‐tissue GHS formation, enabling intra‐particle crosslinking through a Schiff base reaction between primary amines and aldehyde groups. This reaction converts physically crosslinked GelMA microgels to SμG, as schematically shown in Figure 1d. Here, stability refers to the microgel capability in maintaining structural integrity without dissolving at the physiological temperature. SμG subsequently undergoes packing and inter‐particle crosslinking via photocrosslinking, forming GHS^SμG^.

### 3.2 Trade‐off between *D*_S_ and microgel stability

**FIGURE 2:**
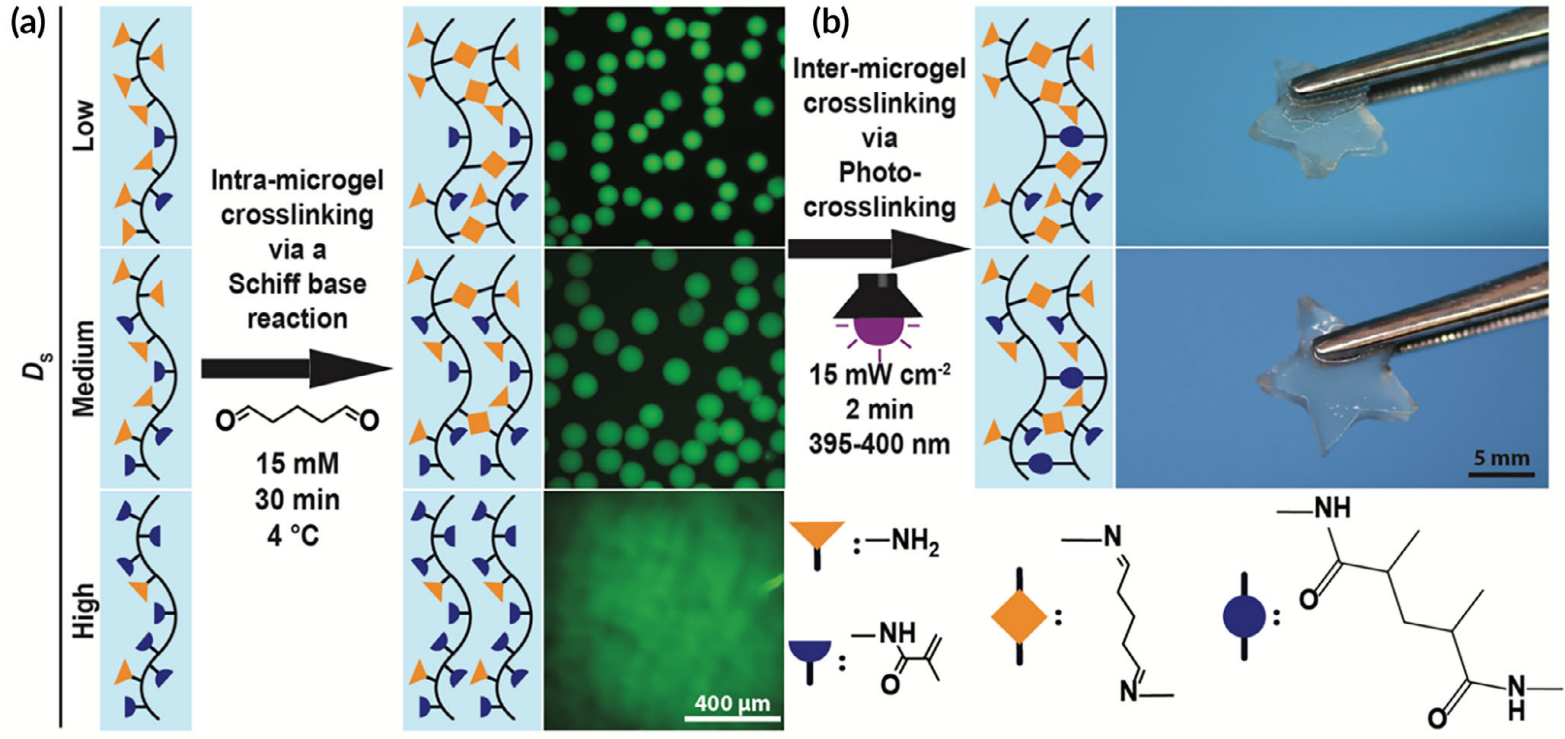
Trade‐off between *D*_S_ and microgel stability. (a) Intra‐particle crosslinking via a Schiff base reaction using GA at 4°C. Unlike high‐*D*_S_ GelMA microgels, low‐ or medium‐*D*_S_ GelMA has enough primary amine groups to form intra‐microgel imine bonds, yielding stable microgels at the physiological temperature (stable microgels [SμG]). (b) Packing the SμG, followed by photocrosslinking (395–400 nm wavelength, 15 mW cm^−2^ intensity, and 2 min exposure time), yields stable GHS^SμG^. Macroscopic images of molded GelMA GHS^SμG^, showing a better shape fidelity in the medium‐*D*_S_ scaffold and minor surface cracks in the low‐*D*_S_ counterpart.

Since primary amines participate in both methacrylation (quantified by $D_{S}$) and intra‐particle Schiff base reaction, there exists a trade‐off between *D*_S_ and microgel stability. Figure 1e qualitatively shows that when *D*_S_ is high, the microgels lack sufficient primary amine groups to form a Schiff base, thus remain unstable at the physiological temperature. Intra‐particle crosslinking via a Schiff base reaction with GA (0.015 mmol mL^−1^) at 4°C renders microgels stable at medium or low *D*_S_. As shown in Figure 2a, GelMA microgels with low or medium *D*_S_ retain enough primary amines for efficient Schiff base formation, resulting in SμG at 37°C; however, the microgels with high *D*_S_ dissolve at the physiological temperature as a result of insufficient intra‐microgel imine bond formation. The corresponding size analysis for low‐ and medium‐*D*_S_ SμG is provided in Figure S3. The smaller microgel diameter in the low‐*D*_S_ group may be a result of higher amine functional group availability for the Schiff base reaction, which results in a higher degree of crosslinking and a lower equilibrium swelling ratio. The SμG fabricated using GelMA with low or medium *D*_S_ are subsequently packed and photocrosslinked (395–400 nm wavelength, 15 mW cm^−2^ intensity, and 2 min exposure time), forming stable GHS^SμG^ (Figure 2b). While both GHS^SμG^ are mechanically robust enough to be handled with tweezers, minor surface cracking is observed in the low‐*D*_S_ constructs. Accordingly, medium‐*D*_S_ GelMA is more suitable for fabricating stable GHS^SμG^ with decent shape fidelity and structural integrity.

To identify optimal conditions for forming SμG under physiological conditions, physically crosslinked medium‐*D*_S_ GelMA microgels are reacted with GA for varying reaction times (0, 2, 5, 15, and 30 min) and subsequently maintained at 37°C to assess their stability (Figure S4a). GA reaction time strongly influences microgel stability; non‐crosslinked microgels dissolve completely within 10 min at 37°C, whereas microgels crosslinked for 30 min are stable over extended incubation periods (e.g., 10^3^ min), with no significant change in diameter compared to their initial state (Figure S4b). Note that the microgels reacted with GA for 2‐15 min undergo significant swelling.

### 3.3 Scaffold pore structure and connectivity

Interconnected pores inherently emerge from the interstitial void spaces formed among the microgels in GHS. Recent studies highlight that void volume fraction alone is insufficient to fully characterize scaffold porosity, necessitating additional descriptors for comprehensive pore analysis.^11^ Therefore, despite GelMA GHS^SμG^ having a comparable void fraction (~22 ± 4%) to our previously reported values for GelMA GHS,^17^, ^18^ further pore characterization is necessary. Beyond geometric description, pore fraction plays a key role in determining GHS integrity and performance,^3^, ^13^ for example, increasing void fraction may reduce inter‐microgel contact area per unit volume and mechanically weaken the scaffold, whereas decreasing void fraction via increasing packing density may increase scaffold stiffness.^13^ Additionally, the void fraction and microarchitectural features of GHS play a central role in regulating mass transport and biological interactions. Large, interconnected pores facilitate nutrient diffusion, cell infiltration, and tissue ingrowth, whereas smaller pores impose physical confinement that can modulate cell structure and function.^3^

**FIGURE 3:**
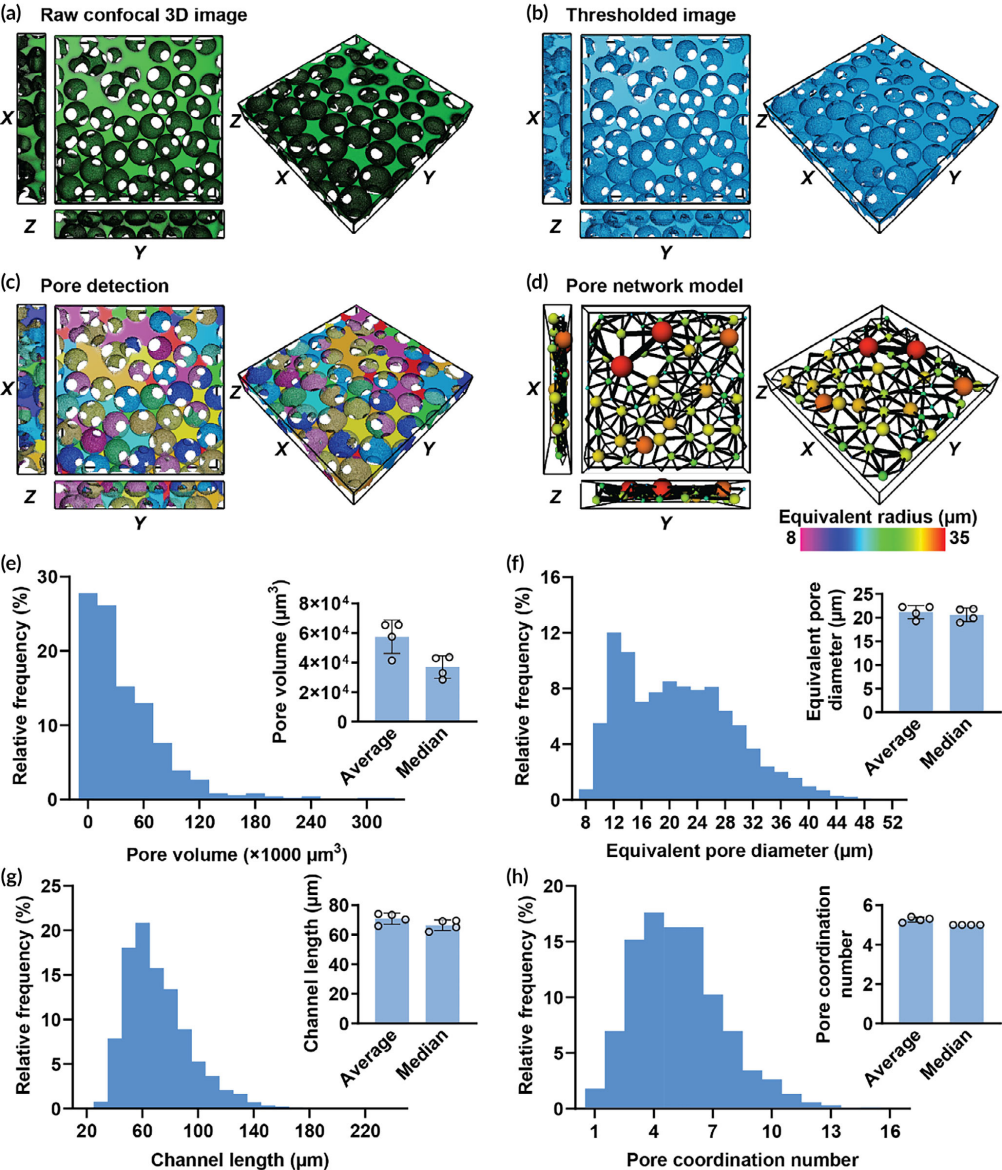
Pore analysis of GelMA GHS^SμG^. (a) Confocal microscopy images showing the 3D pores of GHS^SμG^. (b) Thresholded (binarized) image for pore characterization. (c) 3D pore detection and segmentation of GHS^SμG^. (d) PNM showing interconnectivity, with color‐coded pore radii. Region of interest size is ~500 × 500 × 75 μm^3^. Quantification of GHS^SμG^ pore features, including (e) pore volume distribution, (f) equivalent pore diameter, (g) channel length between pores, and (h) coordination number (number of connections per pore).

In previous studies by us^13^, ^17^, ^36^ and others,^12^, ^37^ pore analysis relied on a 2D slice, where the detected void area was converted to equivalent circles, enabling the measurement of pore diameter distribution. However, this 2D method does not fully describe important pore features, such as geometry, interconnectivity, and channel characteristics, which are all detectable in 3D. Here, confocal microscopy image acquisition followed by 3D image rendering are performed to visualize GHS^SμG^ pores (Figure 3a). After thresholding and binarizing confocal images (Figure 3b), pores are identified using 3D pore detection based on the Chamfer method, as shown in Figure 3c. In Figure 3d, detected pores are represented as spheres with equivalent volumes, establishing a PNM to quantitatively characterize pore size and connectivity through channels (throats) between adjacent pores. The pore volume distribution is shown in Figure 3e, and the equivalent pore diameter distribution is presented in Figure 3f. Median equivalent pore diameter, measured in 3D, is ~21 ± 1 μm, close to the previously reported 2D value of ~20 ± 2 μm.^17^, ^18^ Median channel length between pores is ~67 ± 4 μm, with an average length of ~71 ± 4 μm (Figure 3g), which are comparable to the diameter of individual microgels (~74 ± 4 μm). Coordination number analysis using PNM quantifies pore connectivity by counting the number of connections per pore (Figure 3h). PNM shows that pore coordination numbers range from ~1 to ~13, with a median of 5, indicating a connected 3D pore network. The resulting pore size distribution is not perfectly uniform (Figure 3e,f); however, such polydispersity is an intrinsic feature of randomly packed microgels, which is widely observed in GHS,^38^ and is not a fabrication limitation. Importantly, GHS pore architecture offers key advantages, such as facilitating nutrient diffusion, cell infiltration, and tissue ingrowth. For applications requiring narrower or more uniform pore sizes, several strategies may be implemented, such as using highly monodisperse microgels^38^ and/or controlled or field‐assisted microgel assembly (e.g., via magnetic‐ or template‐assisted packing).^39^, ^40^

### 3.4 Mechanical and rheological properties

**FIGURE 4:**
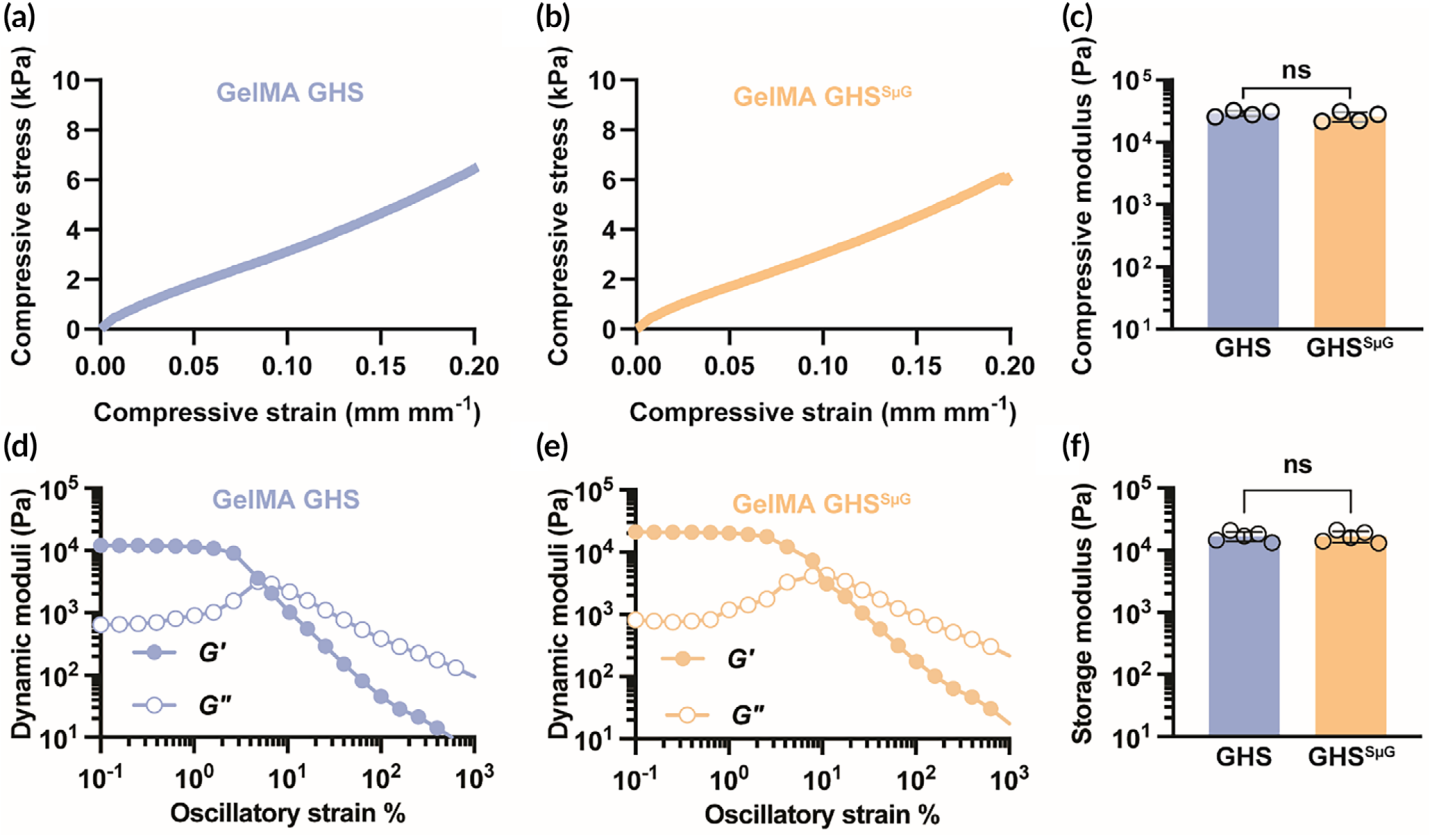
Mechanical and rheological properties of GelMA GHS^SμG^ compared with conventional GHS. Compressive stress–strain curves of (a) conventionally fabricated GelMA GHS and (b) SμG‐based GelMA GHS (GHS^SμG^), showing comparable mechanical performance under compression. (c) Compressive modulus comparison between GHS and GHS^SμG^, indicating no significant difference. Dynamic moduli of (d) GHS and (e) GHS^SμG^ versus oscillatory strain, measured at oscillatory frequency of ~1 rad s^−1^. (f) Storage modulus for GHS and GHS^SμG^. Unpaired two‐tailed Student's *t*‐test is performed (ns denotes *p* ≥ 0.05).

The mechanical and rheological properties of GHS^SμG^, fabricated using the medium‐*D*_S_ GelMA biopolymer, are evaluated via uniaxial compression and oscillatory rheology tests, respectively, and compared with those of GHS, formed using physically crosslinked microgels. Representative compressive stress–strain curves of GelMA GHS (Figure 4a) and GHS^SμG^ (Figure 4b) show comparable mechanical behavior. The compressive modulus, determined using the linear elastic region (within ~0.05–0.15 mm mm^−1^ strain), shows no significant difference between GHS^SμG^ and conventionally fabricated GHS (Figure 4c). The comparison between the mechanical properties of GHS and GHS^SμG^ shows that the Schiff base crosslinking does not disrupt the covalent interlinking of microgels.

To assess viscoelastic properties, oscillatory shear strain sweep tests are conducted on both scaffold types at 25°C (Figure 4d,e) and at 37°C (Figure S5a,b). Figure 4d,e present the dynamic moduli versus oscillatory strain at 25°C for GHS and GHS^SμG^, and Figure S5a,b show the corresponding behavior at 37°C. The average *G*′, measured at 1 rad s^−1^ and 0.1% strain (Figures S5c and S5C), shows no significant differences between GHS and GHS^SμG^ at either temperature. At 25°C (Figure 4f), GHS and GHS^SμG^ have comparable average *G′* values of 17.0 ± 2.8 and 16.7 ± 3.4 kPa, respectively, and at 37°C, the average *G′* is 23.7 ± 5.8 and 22.7 ± 7.8 kPa for GHS and GHS^SμG^, respectively (Figure S5c). A notable difference in the rheological properties of GHS and GHS^SμG^ at 25°C and 37°C is the flow point. The average strain at the flow point of GHS and GHS^SμG^ at 25°C is ~9 ± 1% and ~10 ± 2%, respectively, and at 37°C is ~1.9 ± 0.7% and ~1.6 ± 0.4%, respectively. This reduction may be attributed to increased polymer chain mobility at the physiological temperature.^41^

### 3.5 GelMA GHS and GHS^SμG^ enzymatic degradation

**FIGURE 5:**
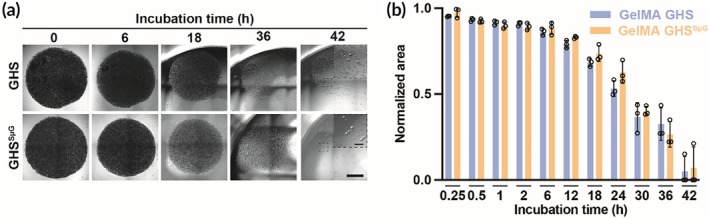
GHS and GHS^SμG^ enzymatic degradation. (a) Brightfield images of GHS and GHS^SμG^, incubated at 37°C in a collagenase solution (5 U mL^−1^) for varying periods. The insets show the remaining microgels at 42 h. (b) Scaffold area at different timepoints, normalized with the initial area (at time 0). Both scaffold types show comparable degradation kinetics and almost fully degrade after ~42 h. Scale bar for (a) is 1 mm, and for the inset is 200 μm.

The enzymatic degradation of gelatin‐based biomaterials is a critical design parameter, governed by the presence of MMP‐sensitive peptide motifs within the gelatin backbone.^3^, ^8^, ^31^ GelMA GHS and GHS^SμG^ are designed to leverage this property, thus collagenase‐mediated scaffold degradation is conducted to compare the degradation kinetics of these scaffolds. Scaffolds were incubated in a collagenase solution (5 U mL^−1^) at 37°C, and their degradation was monitored over time. As shown via brightfield images in Figure 5a, both scaffold types undergo gradual degradation, reflected in area reduction over time, and eventually dissolve in ~42 h. A quantitative assessment of the scaffold area reduction shows no statistically significant difference in the degradation rates between GHS and GHS^SμG^ (Figure 5b). Additionally, the size analysis of remaining microgels at the initial time point and 42 h after incubation in the collagenase solution shows a significant reduction in microgel diameter (Figure S6a,b). The average initial SμG diameter at time 0 is ~76 ± 6 μm, which decreased to ~53 ± 12 μm after 42 h (Figure S6c). Accordingly, the Schiff base reaction within SμG which provides structural stability at the physiological temperature does not significantly affect the enzymatic sensitivity of GelMA GHS^SμG^; however, the significant difference in microgel diameter between GHS and GHS^SμG^ at 42 h suggests that the Schiff base crosslinking may slow down the degradation of residual SμG fraction after the complete disintegration of GHS.

### 3.6 In vitro assessments of cell viability and metabolic activity

**FIGURE 6:**
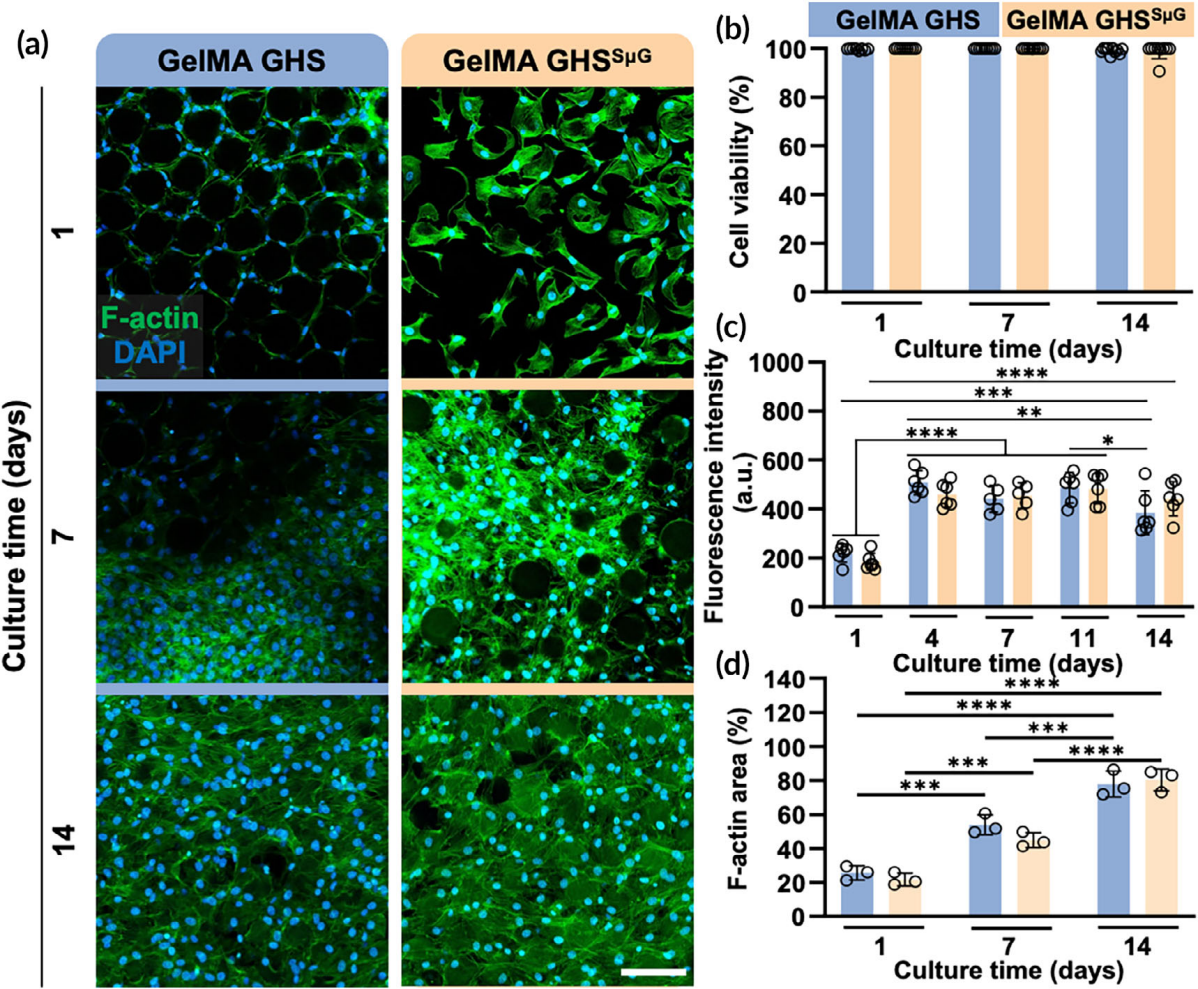
In vitro toxicity assessment of GHS^SμG^ compared with conventional GHS. (a) Fluorescence microscopy images of NIH/3T3 murine fibroblast cells, cultured in the scaffolds and stained with F‐actin (green, cytoskeleton) and DAPI (blue, nuclei), showing cell morphology and proliferation on days 1, 7, and 14 after seeding. In these experiments, cells are topically seeded on the GelMA GHS or GelMA GHS^SμG^. (b) Cell viability quantification (live–dead fluorescence images are presented in Figure S7), showing consistently high viability in all GHS groups within 2 weeks of culture, with no statistically significant differences among the study groups. (c) The metabolic activity of cells, cultured in GHS and GHS^SμG^. (d) The average cell coverage, quantified using F‐actin area on days 1, 7, and 14 after seeding. The scale bar is 100 μm. Ordinary two‐way ANOVA is performed, followed by Tukey's post‐hoc multiple comparison test (*p* ≥ 0.05 is ns, which is not shown for clarity; *p < 0.05, **p < 0.01, ***p < 0.001, and ****p < 0.0001).

The in vitro toxicity of GHS^SμG^ is assessed by evaluating cell viability and metabolic activity over 14 days. GHS and GHS^SμG^ are topically seeded with NIH/3T3 murine fibroblast cells. Fluorescence microscopy images of cell cytoskeleton and nuclei, stained using F‐actin and DAPI, respectively, are acquired on days 1, 7, and 14 post‐seeding (Figure 6a), which show cell spreading and proliferation on both scaffold types, with no observable differences in cell morphology and density between GHS and GHS^SμG^ over time. Figure S7 presents the fluorescence images of stained cells with the viability assay on days 1, 7, and 14 post‐seeding in which live (green) and dead (red) cell staining show minimal cell death in GHS^SμG^, comparable to that in the conventional GHS. Figure 6b presents the viability quantification of cultured cells in GHS and GHS^SμG^ on days 1, 7, and 14 post‐seeding, which consistently shows high viability (~100%) across all scaffold conditions. Metabolic activity measurements (Figure 6c) further indicate an approximately two fold increase from day 1 to day 4, with no statistically significant difference between GHS^SμG^ and GHS. Although metabolic activity plateaus by day 7, the F‐actin area coverage (Figure 6d) continues to increase after 7 and 14 days of culture, confirming ongoing cell proliferation and spreading in the scaffolds. These findings show that, after extensive washing to remove unreacted GA, GHS^SμG^ are non‐toxic, supporting cell viability and proliferation comparable to their GHS counterpart.

## 4 CONCLUSIONS

GelMA GHS are conventionally fabricated using physically crosslinked microgel building blocks below the sol–gel transition temperature. These microgels dissolve in aqueous solutions at the physiological temperature (37°C), compromising GHS cell‐scale pores. To address this limitation, we introduce a dual crosslinking strategy. First, a GA‐mediated Schiff base reaction converts physically crosslinked GelMA microgels to SμG, preserving their MA groups while rendering them structurally stable at 37°C. Subsequently, MA group photocrosslinking covalently interlinks packed microgels, yielding robust GHS^SμG^. We synthesize GelMA with three *D*_S_, referred to as low (18 ± 3%), medium (36 ± 5%), and high (58 ± 6%). An increase in *D*_S_ reduces primary amine groups while increasing the MA group content, representing a trade‐off between microgel Schiff base formation and photocrosslinking capabilities. Consequently, high‐*D*_S_ microgels lack sufficient free amines for the Schiff base formation with GA, resulting in microgel instability at the physiological temperature. Importantly, low and medium $D_{S}$ provide adequate primary amines for intra‐microgel imine bond formation upon reacting with GA, yielding SμG suitable for scaffold (GHS^SμG^) fabrication. Detailed 3D pore characterization of GHS^SμG^ using PNM show a median pore diameter of ~21 ± 1 μm and channel length of ~67 ± 4 μm. Mechanical and rheological analyses, including compression test and oscillatory rheology, show that GHS^SμG^ have compressive modulus and viscoelastic properties comparable to the conventional GHS, fabricated using physically crosslinked microgels. In vitro experiments with NIH/3 T3 murine fibroblast cells confirm high cell viability and metabolic activity increase in GHS^SμG^ over 14 days, showing safety for biomedical use. Collectively, these findings show that GHS^SμG^ can be fabricated at the physiological temperature, retaining 3D pore interconnectivity, mechanical robustness, and biological performance. This method may expand the translational applications of GelMA‐based porous granular biomaterials in tissue engineering and regenerative medicine via enabling in situ formation without compromising structural or functional properties.

## AUTHOR CONTRIBUTIONS

**Zaman Ataie**: Writing – review & editing, Writing – original draft, Visualization, Validation, Methodology, Investigation, Formal analysis, Data curation. **Nawaf Rajaa M. Alharbi**: Methodology, Data curation, Writing – review & editing. **Angelo Roncalli Alves e Silva**: Data curation, Formal analysis, Writing – review & editing. **Angie Castro**: Formal analysis, Data curation, Writing – review & editing, Validation. **Arian Jaberi**: Formal analysis, Data curation, Writing – review & editing, Validation. **Alexander Kedzierski**: Formal analysis, Data curation, Writing – review & editing. **Aneesh Risbud**: Formal analysis, Data curation, Writing – review & editing. **Roya Koshani**: Methodology, Writing – review & editing. **Amir Sheikhi**: Conceptualization, Formal analysis, Investigation, Writing – original draft, Writing – review & editing, Validation, Supervision, Funding acquisition.

## CONFLICT OF INTEREST STATEMENT

All authors declare that they have no conflicts of interest.

## Supporting information


**Data S1.** Supporting Information.

## Data Availability

All data are available in the main manuscript or Supporting Information S1. Further data that support the findings of this study are available from the corresponding author upon reasonable request.

## References

[1] Daly AC . Granular hydrogels in biofabrication: recent advances and future perspectives. Adv Healthc Mater. 2024;13(25):e2301388. doi:10.1002/adhm.202301388 37317658

[2] Daly AC , Riley L , Segura T , Burdick JA . Hydrogel microparticles for biomedical applications. Nat Rev Mater. 2019;5(1):20‐43. doi:10.1038/s41578-019-0148-6 34123409 PMC8191408

[3] Jaberi A , Xiang Y , Sheikhi A . Multiscale structure–property relationships in gelatin‐based granular hydrogel scaffolds. ACS Macro Lett. 2025;14:1569‐1578. doi:10.1021/ACSMACROLETT.5C00441 41051934 PMC12990769

[4] Feng Q , Li D , Li Q , Cao X , Dong H . Microgel assembly: fabrication, characteristics and application in tissue engineering and regenerative medicine. Bioact Mater. 2022;9:105‐119. doi:10.1016/j.bioactmat.2021.07.020 34820559 PMC8586262

[5] Riley L , Schirmer L , Segura T . Granular hydrogels: emergent properties of jammed hydrogel microparticles and their applications in tissue repair and regeneration. Curr Opin Biotechnol. 2019;60:1‐8. doi:10.1016/j.copbio.2018.11.001 30481603 PMC6534490

[6] Sheikhi A , de Rutte J , Haghniaz R , et al. Microfluidic‐enabled bottom‐up hydrogels from annealable naturally‐derived protein microbeads. Biomaterials. 2019;192:560‐568. doi:10.1016/j.biomaterials.2018.10.040 30530245 PMC6400213

[7] Nichol JW , Koshy ST , Bae H , Hwang CM , Yamanlar S , Khademhosseini A . Cell‐laden microengineered gelatin methacrylate hydrogels. Biomaterials. 2010;31(21):5536‐5544. doi:10.1016/j.biomaterials.2010.03.064 20417964 PMC2878615

[8] Yue K , Trujillo‐de Santiago G , Alvarez MM , Tamayol A , Annabi N , Khademhosseini A . Synthesis, properties, and biomedical applications of gelatin methacryloyl (GelMA) hydrogels. Biomaterials. 2015;73:254‐271. doi:10.1016/j.biomaterials.2015.08.045 26414409 PMC4610009

[9] Kalidindi S , Yi H . Micromolding‐based fabrication of chemically functional and stimuli‐responsive opal microparticles via evaporative deposition–neutralization. ACS Appl Eng Mater. 2023;1(8):2217‐2227. doi:10.1021/ACSAENM.3C00293

[10] Visser CW , Kamperman T , Karbaat LP , Lohse D , Karperien M . In‐air microfluidics enables rapid fabrication of emulsions, suspensions, and 3D modular (bio)materials. Sci Adv. 2018;4(1):eaao1175. doi:10.1126/sciadv.aao1175 29399628 PMC5792224

[11] Riley L , Wei G , Bao Y , et al. Void volume fraction of granular scaffolds. Small. 2023;19(40):2303466. doi:10.1002/smll.202303466 PMC10592564 37267936

[12] Griffin DR , Weaver WM , Scumpia PO , Di Carlo D , Segura T . Accelerated wound healing by injectable microporous gel scaffolds assembled from annealed building blocks. Nat Mater. 2015;14(7):737‐744. doi:10.1038/nmat4294 26030305 PMC4615579

[13] Jaberi A , Kedzierski A , Kheirabadi S , et al. Engineering microgel packing to tailor the physical and biological properties of gelatin methacryloyl granular hydrogel scaffolds. Adv Healthc Mater. 2024;13(25):2402489. doi:10.1002/adhm.202402489 PMC11828485 39152936

[14] Ataie Z , Kheirabadi S , Zhang JW , et al. Nanoengineered granular hydrogel bioinks with preserved interconnected microporosity for extrusion bioprinting. Small. 2022;18(37):2202390. doi:10.1002/smll.202202390 35922399

[15] Shenoy G , Kheirabadi S , Ataie Z , et al. Iron inhibits glioblastoma cell migration and polarization. FASEB J. 2023;37(12):e23307. doi:10.1096/fj.202202157RR 37983646

[16] Jaberi A , Ghelich P , Samandari M , et al. Gelatin methacryloyl granular hydrogel scaffolds for skin wound healing. Biomater Sci. 2025;13:6013‐6023. doi:10.1039/d4bm01062k 40298015 PMC12038805

[17] Ataie Z , Horchler S , Jaberi A , et al. Accelerating patterned vascularization using granular hydrogel scaffolds and surgical micropuncture. Small. 2024;20(8):2307928. doi:10.1002/smll.202307928 PMC11699544 37824280

[18] El‐Mallah JC , Ataie Z , Horchler SN , et al. Micropuncture and granular hydrogel scaffolds to surgically bioengineer a perfusable and stably patterned microvasculature. Angiogenesis. 2025;28(4):1‐15. doi:10.1007/S10456-025-10003-X PMC12423246 40928571

[19] Ataie Z , Jaberi A , Kheirabadi S , Risbud A , Sheikhi A . Gelatin methacryloyl granular hydrogel scaffolds: high‐throughput microgel fabrication, lyophilization, chemical assembly, and 3D bioprinting. J vis Exp. 2022;190(190):e64829. doi:10.3791/64829 36571405

[20] Zoratto N , Di Lisa D , de Rutte J , et al. In situ forming microporous gelatin methacryloyl hydrogel scaffolds from thermostable microgels for tissue engineering. Bioeng Transl Med. 2020;5(3):e10180. doi:10.1002/btm2.10180 33005742 PMC7510466

[21] Xiang Y , Ataie Z , Castro A , Woo KB , Sheikhi A . Generalizing gelatin methacryloyl granular hydrogel fabrication using stable microgels with predictable stiffness. Adv Healthc Mater. 2025;14(31):2500154. doi:10.1002/ADHM.202500154 PMC12596051 40457645

[22] Hellio D , Djabourov M . Physically and chemically crosslinked gelatin gels. Macromol Symp. 2006;241(1):23‐27. doi:10.1002/masy.200650904

[23] Rebers L , Reichsöllner R , Regett S , et al. Differentiation of physical and chemical cross‐linking in gelatin methacryloyl hydrogels. Sci Rep. 2021;11(1):3256. doi:10.1038/s41598-021-82393-z 33547370 PMC7864981

[24] Mugnaini G , Gelli R , Mori L , Bonini M . How to cross‐link gelatin: the effect of glutaraldehyde and glyceraldehyde on the hydrogel properties. ACS Appl Polym Mater. 2023;5(11):9192‐9202. doi:10.1021/acsapm.3c01676

[25] Diba M , Koons GL , Bedell ML , Mikos AG . 3D printed colloidal biomaterials based on photo‐reactive gelatin nanoparticles. Biomaterials. 2021;274:120871. doi:10.1016/j.biomaterials.2021.120871 34029914 PMC8196631

[26] Wang H , Hansen MB , Löwik DWPM , et al. Oppositely charged gelatin nanospheres as building blocks for injectable and biodegradable gels. Adv Mater. 2011;23(12):H119‐H124. doi:10.1002/adma.201003908 21394793

[27] Saeedinejad F , Alipanah F , Toro S , et al. In situ‐formed tissue‐adhesive macroporous scaffolds enhance cell infiltration and tissue regeneration. Acta Biomater. 2025;200:358‐377. doi:10.1016/j.actbio.2025.04.049 40288431

[28] Fang Y , Guo Y , Ji M , et al. 3D printing of cell‐laden microgel‐based biphasic bioink with heterogeneous microenvironment for biomedical applications. Adv Funct Mater. 2021;32:2109810. doi:10.1002/adfm.202109810

[29] Claaßen C , Claaßen MH , Truffault V , et al. Quantification of substitution of gelatin methacryloyl: best practice and current pitfalls. Biomacromolecules. 2018;19(1):42‐52. doi:10.1021/acs.biomac.7b01221 29211461

[30] Schindelin J , Arganda‐Carreras I , Frise E , et al. Fiji: an open‐source platform for biological‐image analysis. Nat Methods. 2012;9(7):676‐682. doi:10.1038/nmeth.2019 22743772 PMC3855844

[31] Yue K , Li X , Schrobback K , et al. Structural analysis of photocrosslinkable methacryloyl‐modified protein derivatives. Biomaterials. 2017;139:163‐171. doi:10.1016/j.biomaterials.2017.04.050 28618346 PMC5845859

[32] Zatorski JM , Montalbine AN , Ortiz‐Cárdenas JE , Pompano RR . Quantification of fractional and absolute functionalization of gelatin hydrogels by optimized ninhydrin assay and 1H NMR. Anal Bioanal Chem. 2020;412(24):6211‐6220. doi:10.1007/s00216-020-02792-5 32617761 PMC7484248

[33] Van Den Bulcke AI , Bogdanov B , De Rooze N , Schacht EH , Cornelissen M , Berghmans H . Structural and rheological properties of methacrylamide modified gelatin hydrogels. Biomacromolecules. 2000;1(1):31‐38. doi:10.1021/bm990017d 11709840

[34] Tosh SM , Marangoni AG , Hallett FR , Britt IJ . Aging dynamics in gelatin gel microstructure. Food Hydrocoll. 2003;17(4):503‐513. doi:10.1016/S0268-005X(03)00018-3

[35] Djabourov M . Architecture of gelatin gels. Contemp Phys. 1988;29(3):273‐297. doi:10.1080/00107518808224377

[36] Kedzierski A , Kheirabadi S , Jaberi A , et al. Engineering the hierarchical porosity of granular hydrogel scaffolds using porous microgels to improve cell recruitment and tissue integration. Adv Funct Mater. 2025;35(12):2417704. doi:10.1002/adfm.202417704

[37] Qazi TH , Muir VG , Burdick JA . Methods to characterize granular hydrogel rheological properties, porosity, and cell invasion. ACS Biomater Sci Eng. 2022;8(4):1427‐1442. doi:10.1021/acsbiomaterials.1c01440 35330993 PMC10994272

[38] Saxena Y , Riley L , Wu R , Kabir MS , Randles A , Segura T . 3D pore shape is predictable in randomly packed particle systems. Matter. 2025;8(12):102493. doi:10.1016/j.matt.2025.102493 PMC12629294 41268054

[39] Rivera‐Llabres VG , Manrique S , Lapish L , et al. Patterning of anisotropic physical cues in granular PEG hydrogel composites using magnetic templating. Ind Eng Chem Res. 2025;64(35):17038‐17048. doi:10.1021/ACS.IECR.5C02231 41613382 PMC12851619

[40] Wang Y , Zhang Y , Guan Y , Zhang Y . Magnetic field‐assisted fast assembly of microgel colloidal crystals. Langmuir. 2022;38(19):6057‐6065. doi:10.1021/ACS.LANGMUIR.2C00297 35502583

[41] Kokol V , Pottathara YB , Mihelčič M , Perše LS . Rheological properties of gelatine hydrogels affected by flow‐ and horizontally‐induced cooling rates during 3D cryo‐printing. Colloids Surf A Physicochem Eng Asp. 2021;616(3):126356. doi:10.1016/j.colsurfa.2021.126356

