# Supplementary material for: Orthogonally crosslinked gelatin methacryloyl microgels for in situ assembly of granular hydrogel scaffolds
Source: Bioeng Transl Med. 2026 Apr 28;11(3):e70127. doi: 10.1002/btm2.70127 (PMC13247424; doi:10.1002/btm2.70127)
Supplement: Supplementary file 1 — Data S1. Supporting Information. [file BTM2-11-e70127-s001.pdf]

Supporting Information

Orthogonally crosslinked gelatin methacryloyl microgels for *in situ* assembly of granular hydrogel scaffolds

Zaman Ataie[1], Nawaf Rajaa M. Alharbi[2], Angelo Roncalli Alves e Silva[1,3], Angie Castro[1], Arian Jaberi[1], Alexander Kedzierski[2], Aneesh Risbud[2], Roya Koshani[1], Amir Sheikhi[1,2,4,5,6]*

[1]Department of Chemical Engineering, The Pennsylvania State University, University Park, PA 16802, USA

[2]Department of Biomedical Engineering, The Pennsylvania State University, University Park, PA 16802, USA

[3]Núcleo de Biologia Experimental, Universidade de Fortaleza, Fortaleza, CE 60811605, Brazil

[4]Department of Chemistry, The Pennsylvania State University, University Park, PA 16802, USA

[5]Department of Neurosurgery, College of Medicine, The Pennsylvania State University, Hershey, PA 17033, USA

[6]Huck Institutes of the Life Sciences, The Pennsylvania State University, University Park, PA 16802, USA

*Corresponding author: Amir Sheikhi (sheikhi@psu.edu)

Supporting Information includes **Figures S1** to **S7**.

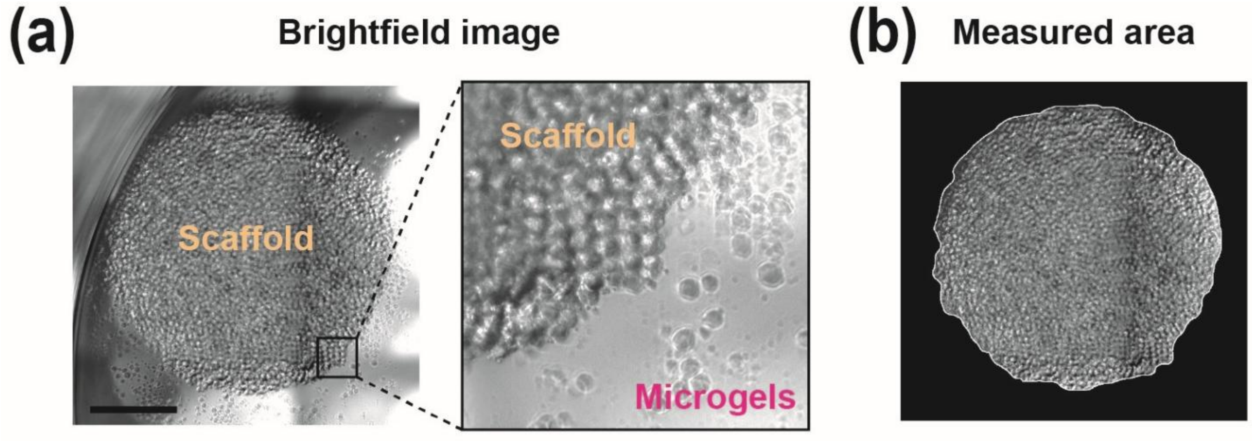

Figure S1. GHS area measurement. (a) Brightfield images of GHS, incubated at 37°C in a collagenase solution (5 U mL^{-1}) for 24 h. Scale bar is 1 mm. The inset shows the edge of GHS and detached microgels. **(b)** Measured scaffold area using ImageJ.

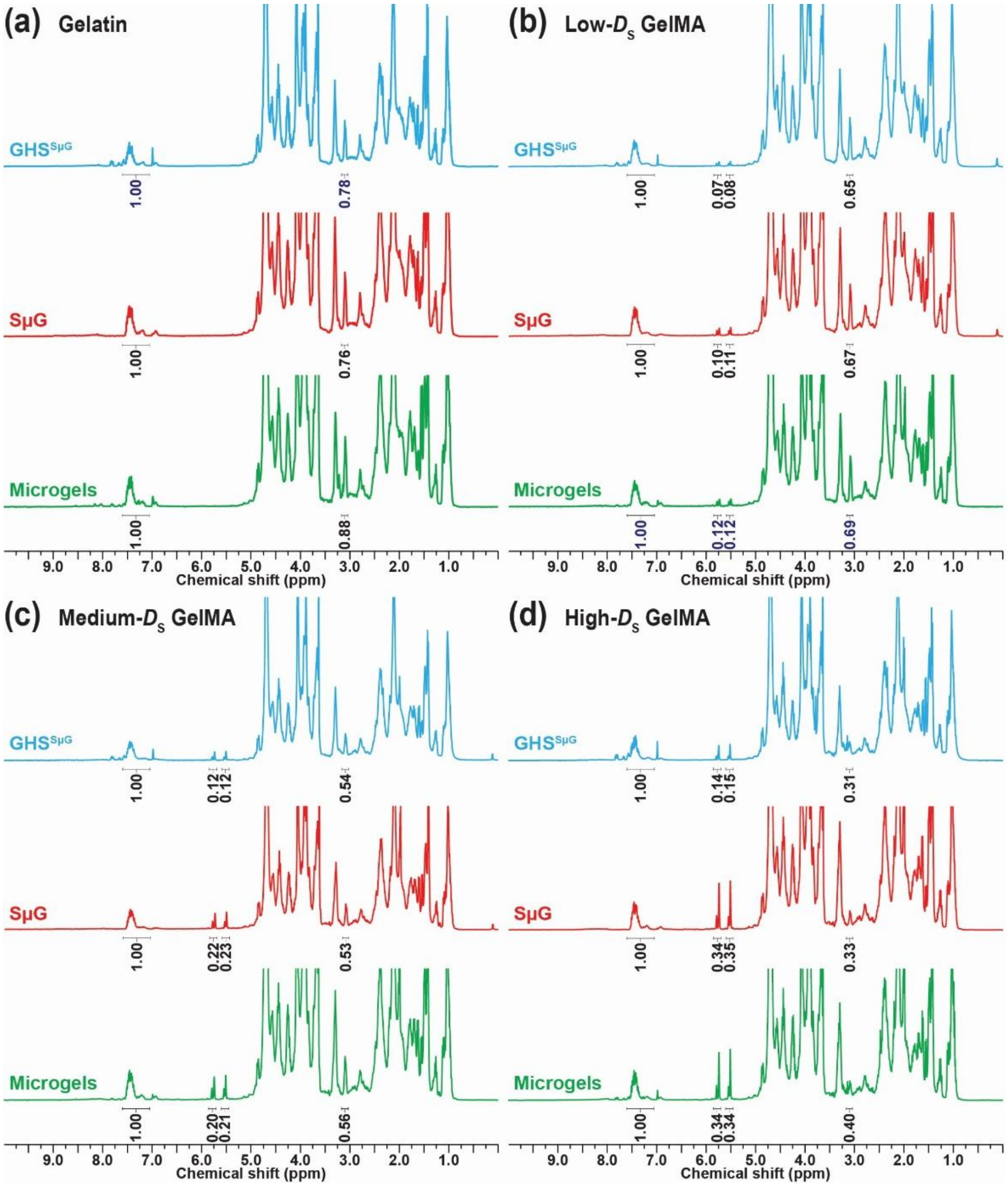

Figure S2. Proton nuclear magnetic resonance (^{1}H NMR) spectroscopy, used to measure GelMA degree of methacryloyl (MA) substitution (*D*S). The ^{1}H NMR spectra of microgels, SμG, and GHS^{SμG}, fabricated using **(a)** gelatin, **(b)** low-*D*S GelMA, **(c)** medium-*D*S GelMA, or **(d)** high-*D*S GelMA. Phenylalanine aromatic groups (~7.0-7.6 ppm) are the internal reference. Lysine methylene protons (~2.8-3.2 ppm) and vinyl protons (~5.3-6.0 ppm) are integrated to calculate the *D*S.

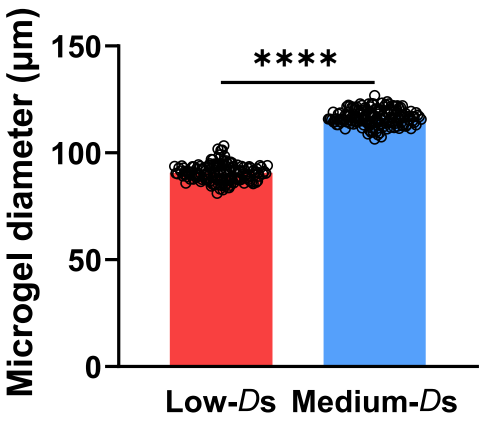

Figure S3. Low- and medium-*D*S GelMA SμG diameter. The smaller microgel diameter in the low-*D*S group compared with the medium-*D*S counterpart may be a result of higher amine availability for the Schiff base reaction, yielding a higher degree of crosslinking and a lower equilibrium swelling ratio. Unpaired two-tailed Student's *t*-test is performed (****$p < 0.0001$).

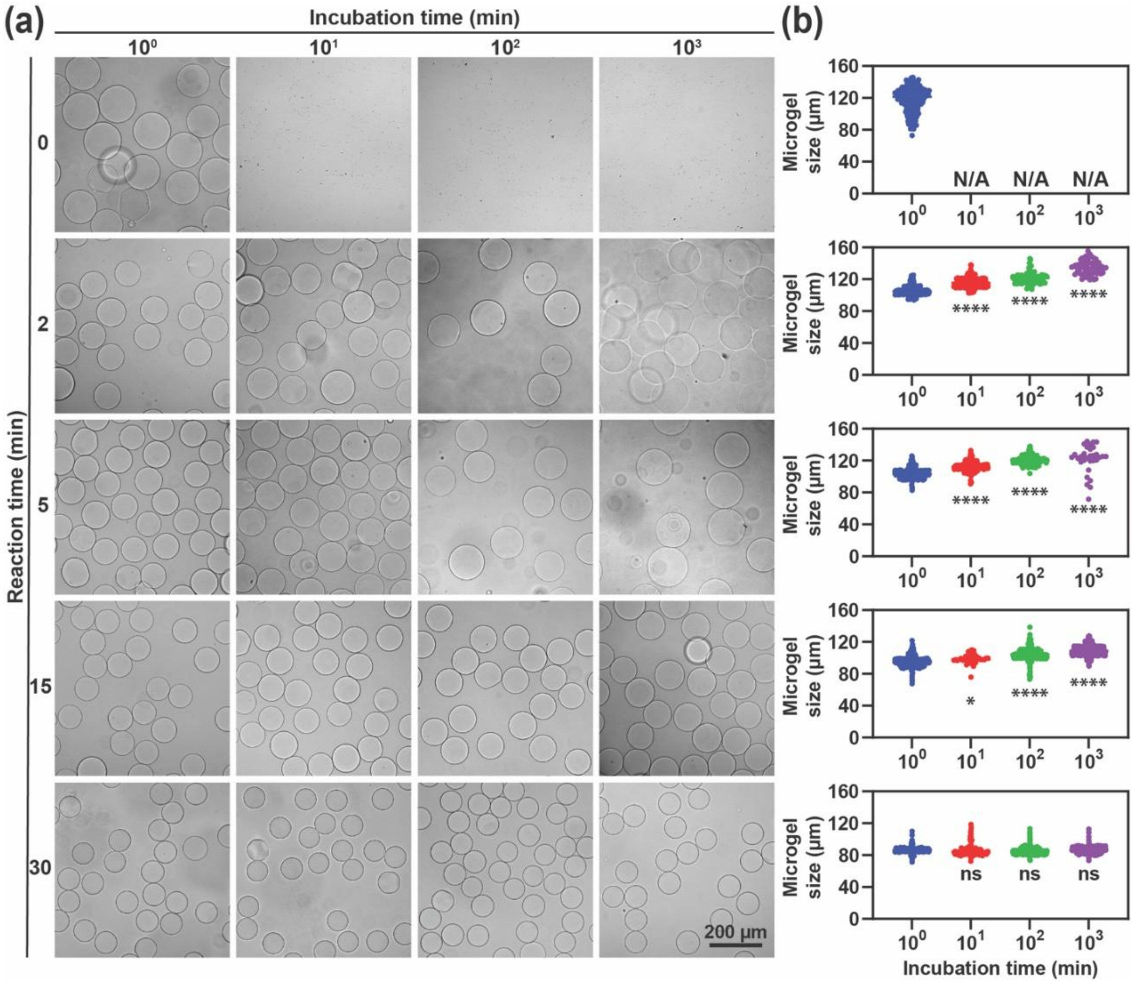

Figure S4. The stability of GelMA microgels after reacting with glutaraldehyde (GA) for varying reaction times. (a) Bright-field micrographs of physically crosslinked GelMA microgels after GA reaction times of 0 (no reaction), 2, 5, 15, and 30 min, followed by incubation at 37°C for varying durations. Non-crosslinked microgels dissolve within ~10 min. **(b)** Microgel size (diameter) distributions, obtained based on three independent experiments with ≥ 30 microgels analyzed per condition. "N/A" indicates complete microgel dissolution. One-way ANOVA is performed, followed by Tukey's post-hoc multiple comparison test. Asterisks denote statistical differences between the size of microgels incubated for 10, 100, or 1000 min at 37°C with the size of those incubated for 1 min at the same condition (ns = not significant, $p \geq 0.05$; *$p < 0.05$, and ****$p < 0.0001$).

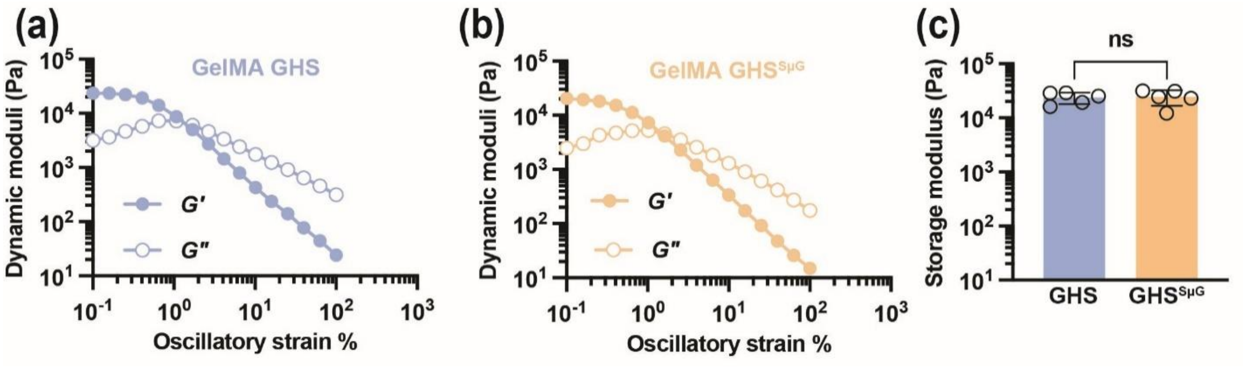

Figure S5. Rheological properties of GelMA GHS$^{S\mu G}$ compared with conventional GHS at 37°C. Dynamic moduli of **(a)** GHS and **(b)** GHS$^{S\mu G}$ versus oscillatory strain, measured at oscillatory frequency of ~1 rad s^{-1}. **(c)** Storage modulus of GHS and GHS$^{S\mu G}$. Unpaired two-tailed Student's *t*-test is performed (ns shows $p \geq 0.05$).

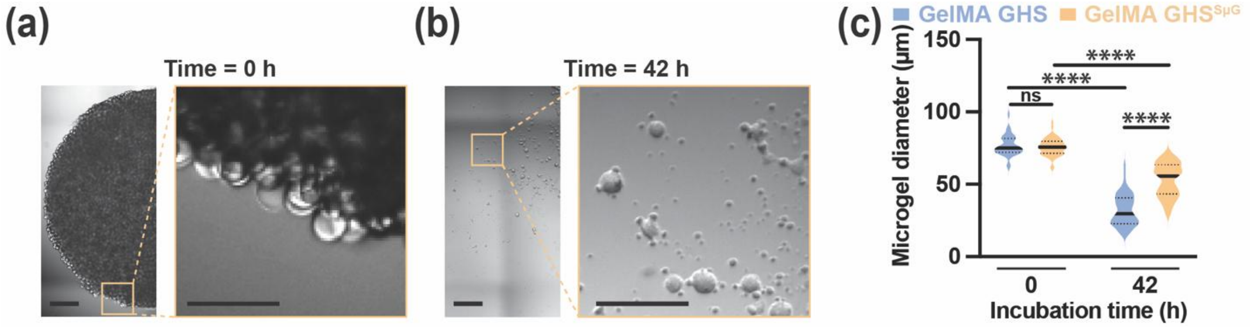

Figure S6. GHS and GHS$^{S\mu G}$ enzymatic degradation. (a) Brightfield images of GHS$^{S\mu G}$ at the initial timepoint, with an inset highlighting microgels at the scaffold edge. **(b)** Brightfield images of GHS$^{S\mu G}$ after 42 h of incubation in a collagenase solution (5 U mL^{-1}) at 37°C, with an inset highlighting microgels released from the scaffold. **(c)** Microgel size (diameter) distribution. Ordinary two-way ANOVA is performed, followed by Tukey's post-hoc multiple comparison test (ns denotes $p \geq 0.05$ and ****$p < 0.0001$; some of the comparisons are not shown for clarity). Scale bars are 200 μm.

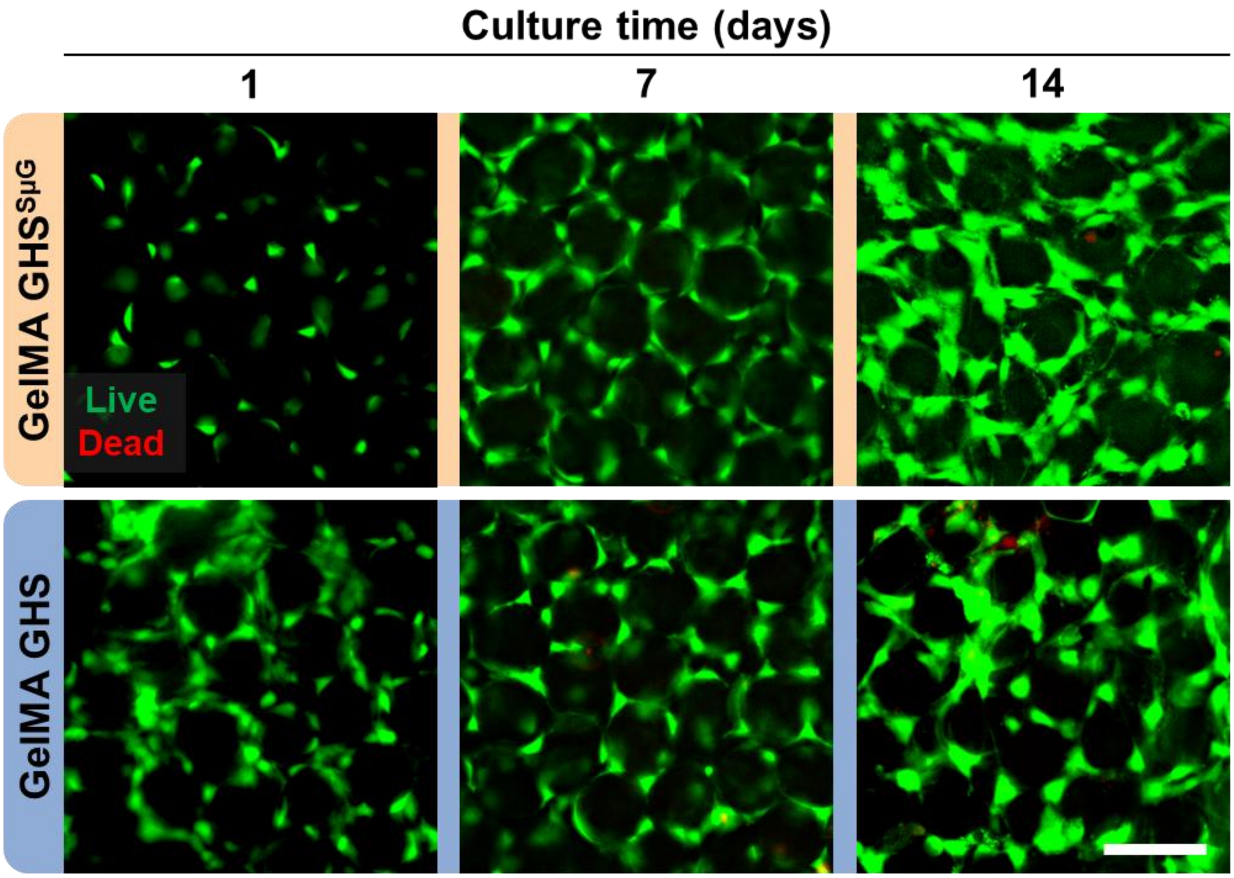

Figure S7. *In vitro* toxicity assessment of GHS$^{S\mu G}$ compared with conventional GHS. Fluorescence microscopy images of NIH/3T3 murine fibroblast cells, cultured in GHS$^{S\mu G}$ or GHS and stained using Calcein AM (representing live cells, green) and BOBO-3 iodide (representing dead cells, red) fluorophores, showing high cell viability and progressive proliferation. In these experiments, cells are seeded on GelMA GHS or GHS$^{S\mu G}$, comprising physically crosslinked or GA-mediated chemically crosslinked microgels, respectively. Scale bar is 100 μm.
